# Graphene’s cousin: the present and future of graphane

**DOI:** 10.1186/1556-276X-9-26

**Published:** 2014-01-13

**Authors:** Chao Zhou, Sihao Chen, Jianzhong Lou, Jihu Wang, Qiujie Yang, Chuanrong Liu, Dapeng Huang, Tonghe Zhu

**Affiliations:** 1 College of Chemistry and Chemical Engineering, Shanghai University of Engineering Science, Shanghai 201620, China; 2 Department of Chemical and Biomedical Engineering, North Carolina A&T State University, 1601 E. Market St, Greensboro NC 27411, USA

**Keywords:** Graphene, Graphane, Partially hydrogenated graphene, Nanostructure

## Abstract

The so-called graphane is a fully hydrogenated form of graphene. Because it is fully hydrogenated, graphane is expected to have a wide bandgap and is theoretically an electrical insulator. The transition from graphene to graphane is that of an electrical conductor, to a semiconductor, and ultimately to an electrical insulator. This unique characteristic of graphane has recently gained both academic and industrial interest. Towards the end of developing novel applications of this important class of nanoscale material, computational modeling work has been carried out by a number of theoreticians to predict the structures and electronic properties of graphane. At the same time, experimental evidence has emerged to support the proposed structure of graphane. This review article covers the important aspects of graphane including its theoretically predicted structures, properties, fabrication methods, as well as its potential applications.

## Review

**Figure 1:**
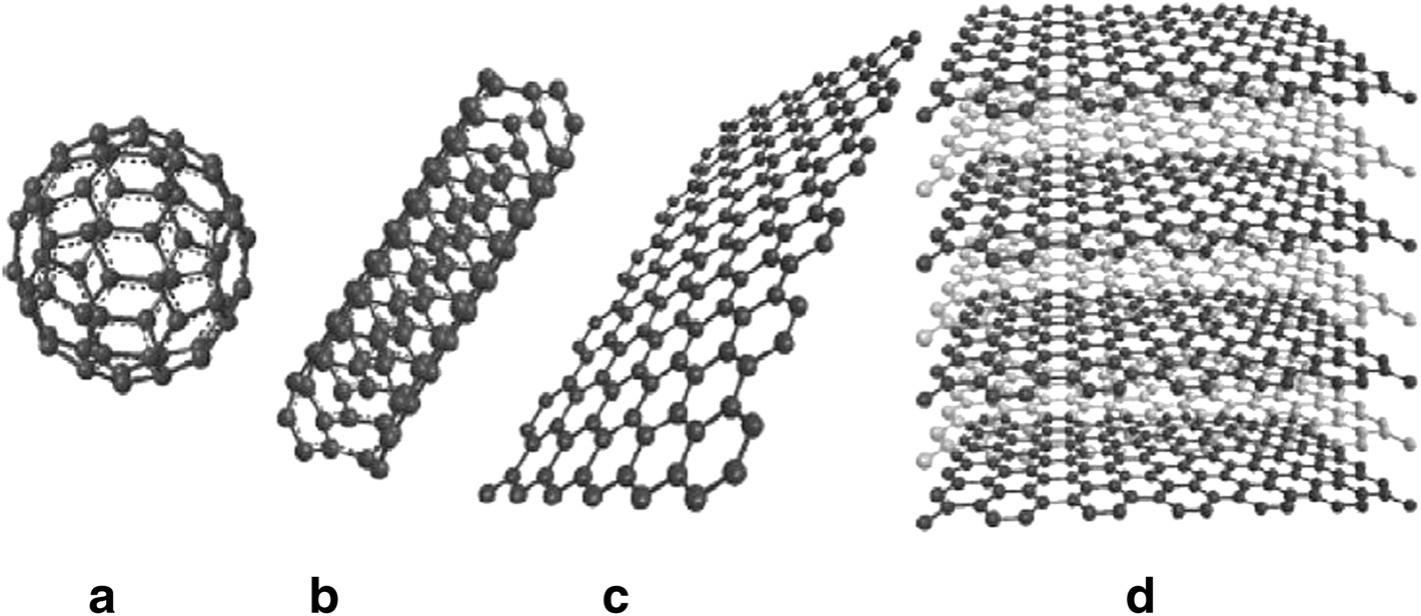
**Forms of *sp***^**2**^**-bonded carbon. (a)** Fullerene (0D), **(b)** single-walled carbon nanotubes (1D), **(c)** graphene (2D), **(d)** graphite (3D) [35].

Graphene was first discovered in 2004 by Novoselov et al. [1]. Graphene is a single atomic layer with a thickness of only 0.34 nm of *sp*^2^ hybridized carbon atoms covalently bonded to three other atoms arranged in a honeycomb lattice [1-7]. Graphene's unique structural, mechanical, and electrical properties and high carrier mobility makes it one of the most important topics in materials science today [8-14]. Graphene forms the basic structure of other carbon-based materials such as fullerene (wrapped-up graphene) [15-21], carbon nanotubes (several graphene sheets rolled up along a vertical axis) [22-29], and graphite (stacked graphene) [30-35]. Some of the carbon-based materials are illustrated in Figure 1.

Graphene has unique properties with tremendous potential applications, such as chemical sensors [36,37], nanoelectronic devices [38], hydrogen storage systems [39], or polymer nanocomposites [40]. Graphene could be considered as a prototypical material to study the properties of other two-dimensional nanosystems. Several two-dimensional structures have been explored in the literature [41,42]. Graphene-like two-dimensional silicon carbide [43,44], silicon [45,46], germanium [47,48], boron nitride [49,50], and zinc oxide [51] have been explored in the literature.

**Figure 2:**
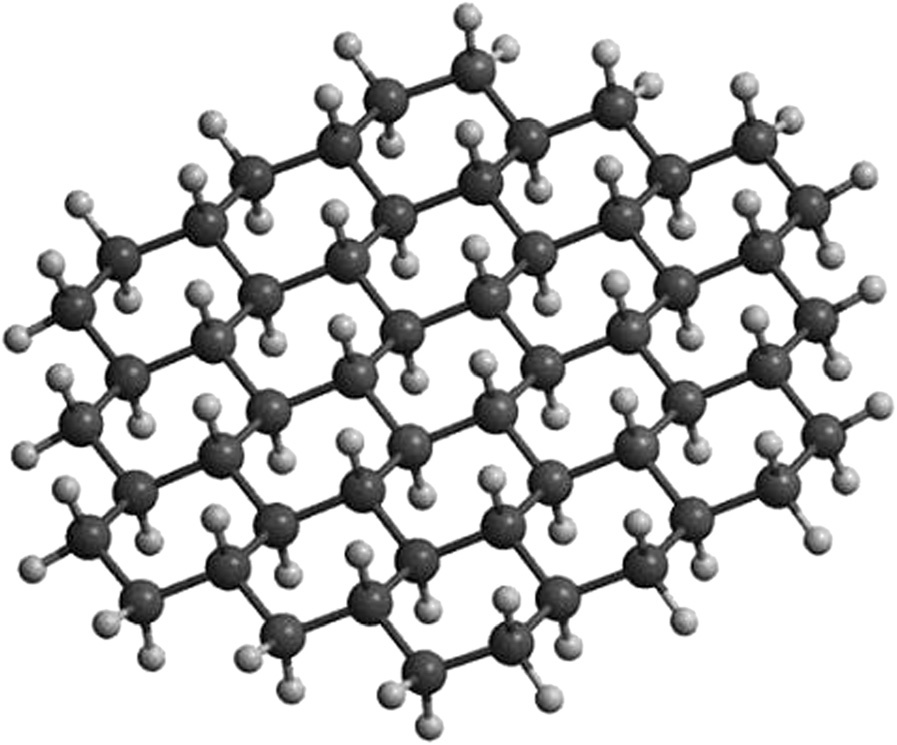
**The diagram of graphane layer **[41]**.**

One important development since the discovery of graphene is the discovery of the so-called graphane, which is a fully hydrogenated form of graphene, as shown in Figure 2. In this form, all carbon atoms in this fully hydrogenated form assume in the *sp*^3^ hybridization. This novel material, graphane, was first proposed by Lu et al. in theoretical investigation [41], and the predicted graphane structure was later confirmed by an experiment by Elias et al. [42]. It was reported that graphene was changed into a new structure called graphane by exposing graphene to hydrogen plasma for several hours. Graphane is predicted to be a stable structure consisting of a graphene layer in which each C atom is *sp*^3^-bonded to one H atom above and below the C atom in an alternating manner [52]. Graphane is predicted to have a bandgap of about 3.5 eV and has potential applications in electronics. In addition to forming graphane, hydrogen plasma exposure was observed to form partially hydrogenated graphene, which consisted of a graphene layer in which only one side was hydrogenated. Although hydrogenation of only one side of graphene is not predicted to be stable, it is proposed that ripples in graphene, which have *sp*^3^-like bonding angles, facilitate the *sp*^3^ bonding of C with H on only one side of the graphene. Partially hydrogenated graphene is observed to be insulating and thus has potential applications in electrical isolation for graphene-based circuits [53].

This review article is intended to focus on the fabrication and structure features of graphane (or graphane-like [54,55]) and the potential application of graphane (or graphane-like) and properties. It covers the latest developments and new perspectives of graphane-based hydrogen storage [56] and transistor [57] with the special discussions on the merits and limitations of the material. Except for presenting a brief overview of the synthesis processes of single-layer graphane, graphane-like, graphene-graphane, graphane nanoribbons [58,59], respectively, the structure features of graphane, particularly related to hydrogen storage and transistor, have been discussed.

### Computational modeling of graphane

**Figure 3:**
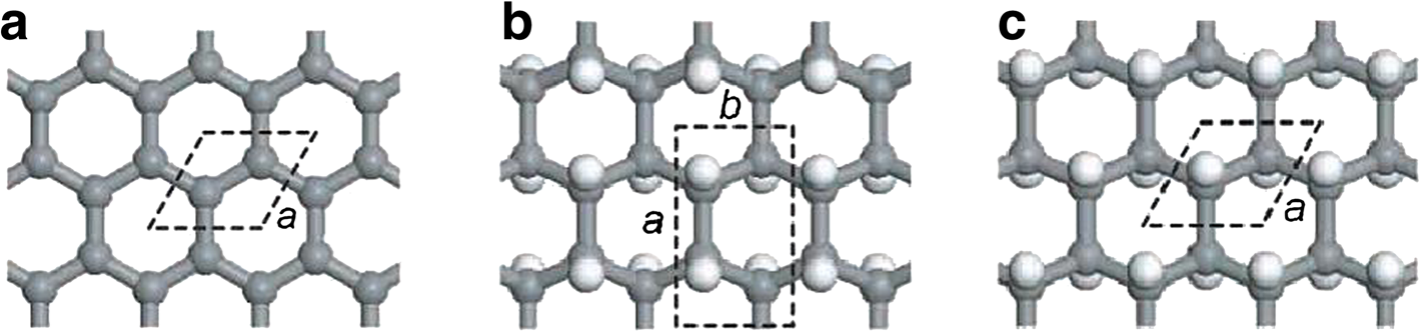
**Structural carbon membrane models considered in DMol3 geometry optimization calculations. (a)** Graphene, having two atoms per unit cell; **(b)** graphane boat-like, with four carbon atoms and four hydrogen atoms per unit cell; **(c)** graphane chair-like, with four (two C and two H) atoms per unit cell. The dashed lines indicate the corresponding unit cell. **(a)** and **(b)** refer to the lattice parameters [60].

Flores et al. [60] used *ab initio* quantum calculations in order to optimize the geometry of graphane-like structures. They used classical reactive bond-order approach in order to investigate the effects of hydrogenation on geometrical structures for a number of graphene membrane models. Molecular dynamics (MD) simulations were used to address the dynamics of hydrogen incorporation into graphene membranes. As the results are displayed, H frustration were very likely to occur, perfect graphane-like structures are unlikely to be formed, and hydrogenated domains are very stable (relevant parameter and crystalline structures shown in Table 1 and Figure 3).

**Table 1:** **Predicted energy per atom in unit cell, cell parameter values, and carbon-carbon distances for graphene and chair-like and boat-like graphane, respectively **[60]

		**Graphene**	**G-chair**	**G-boat**
Energy (Ha) (1 Ha = 27.211 eV)		-304.68	-309.41	-309.38
Lattice parameters:	*a* (Ǻ)	2.465	2.540	4.346
	*b* (Ǻ)	2.465	2.540	2.509
	*γ* (^。^)	120	120	90
C-C bond length (Ả)		1.423	1.537	1.581, 1.537

Note, lattice constant (or called the lattice constant) means the cell length, namely each parallelepiped unit side, he is the crystal structure of an important basic parameters.

Dora et al. [61] used density functional theory, which studies the density of states in monolayer graphene (MLG) and bilayer graphene (BLG) at low energies in the presence of a random symmetry-breaking potential. And it had a breaking potential, which opens a uniform gap, and a random symmetry-breaking potential also created tails in the density of states.

### Experimental synthesis of graphane

The transition from graphene to graphane is that of an electrical conductor to a semiconductor and ultimately to an insulator, which is dependent upon the degree of hydrogenation.

In 2009, the graphane was synthesized by exposing the single-layer graphene to a hydrogen plasma [42].

**Figure 4:**
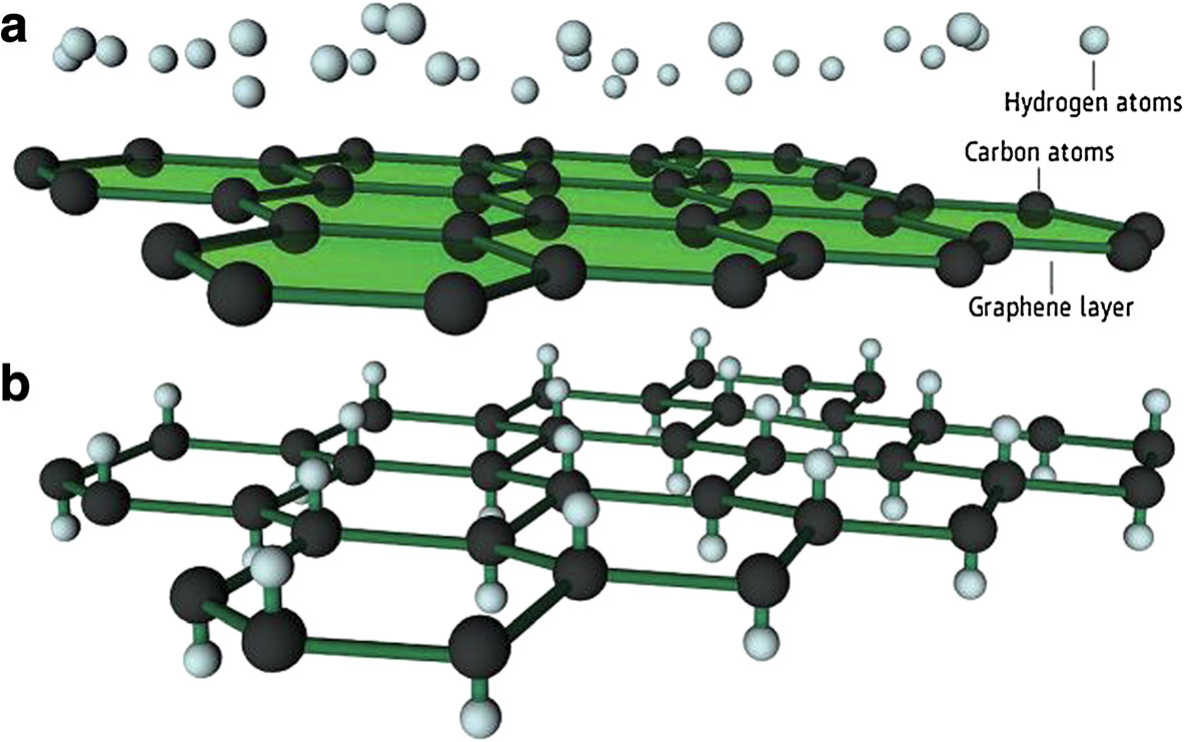
**Graphene hydrogenation progress. (a)** A graphene layer, where delocalized electrons are free to move between carbon atoms, is exposed to a beam of hydrogen atoms. **(b)** In nonconductive graphane, hydrogen atoms bond to their electrons with electrons of carbon atoms and pull the atoms out of the plane [57].

Savchenko [57] used hydrogen plasma to react with graphene for the preparation of graphane and the preparation process was shown in Figure 4. This method was not able to control the degree of hydrogenation.

**Figure 5:**
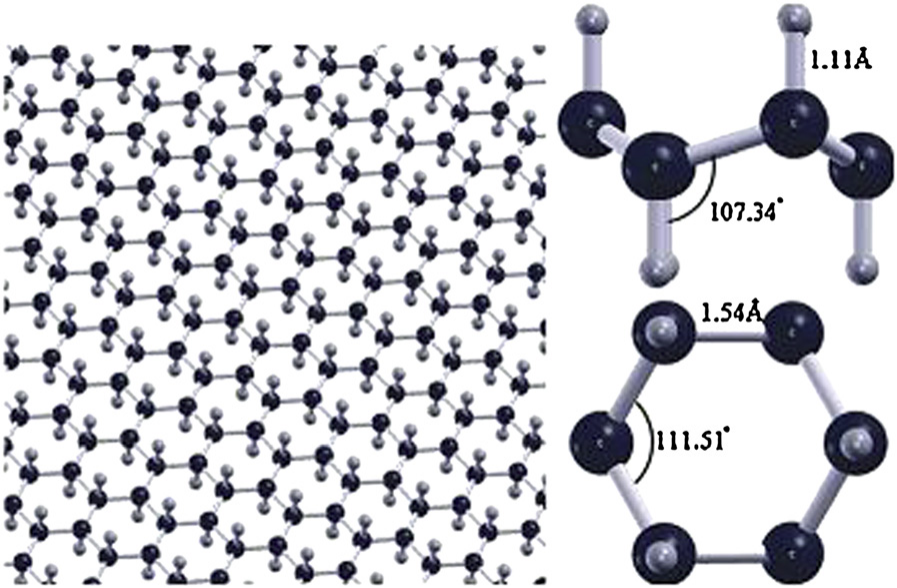
**Structure of graphane (left) and graphane molecule side and top views (right) **[62]**.**

Wang et al. [62] reported a new route to prepare high-quality and monolayer graphane by plasma-enhanced chemical vapor deposition (the structures model as shown in Figure 5). A large-area monolayer graphane-like film was produced by remote-discharged radio frequency plasma beam deposition at 650°C on Cu/Ti-coated SiO_2_-Si. The advantages of the plasma deposition were very short deposition time (<5 min) and very low growth temperature of 650°C compared to the current thermal chemical vapor deposition approach (1,000°C).

### Structures of graphane

**Figure 6:**
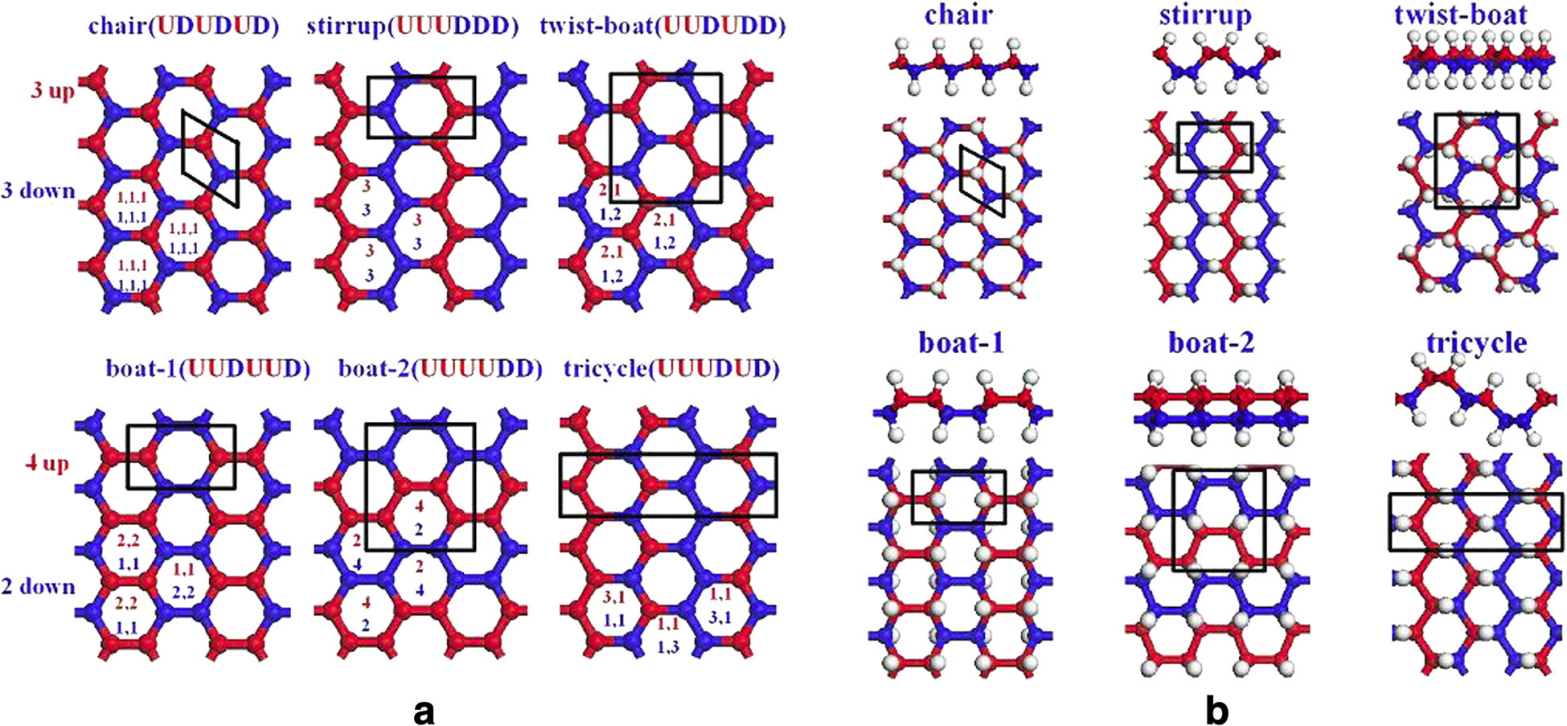
**Schematic diagram of six possible hydrogenated graphene configurations (a) and graphane crystal structures (b). (a)** Configurations with equivalent hexagonal hydrocarbon rings. **(b)** side and top views of graphane crystal structure with chair, stirrup, twist-boat, boat-1, boat-2, and tricycle configurations, respectively. The red and blue balls correspond to carbon atoms with up and down hydrogenation, respectively, and the white balls are hydrogen atoms [70].

Many configurations with low energies for graphane were proposed. Sluiter et al. [63] and Sofo et al. [64] reported that the most stable configuration of graphane was the chair-like structure, with the UDUDUD hydrogenation in each hexagonal carbon ring as shown in Figure 6a [65]. Sluiter et al. [63], Leenaerts et al. [66], and Bhattacharya et al. [67] reported that the second stable configuration was the 'stirrup’ with the UUUDDD hydrogenation in each carbon ring shown in Figure 6a, whose energy was about 28 meV/atom larger than that of the chair one. At the point of stability, the following configurations for graphane allotropes are boat-1 [63,64,66] with the UUDDUU hydrogenation, boat-2 [65,66] with the UUUUDD hydrogenation, twist-boat [68] with the UUDUDD hydrogenation and other configurations with relatively high energies which were reported in the literatures [65,69]. Recently, He et al. [70] used the restrictive condition of keeping the hexagonal hydrocarbon rings equivalent in the systems, and proposed a tricycle graphane allotrope in which each hexagonal hydrocarbon ring with the same UUUDUD hydrogenation was equivalent, as shown in Figure 6b. Table 2 summarizes the structure information for the six fundamental allotropes of graphane [70].

**Table 2:** Structure information

**System**	**SG and LC**	**Positions**	**LCH and LCC**
Chair	P-3 m1 (164),	H: (0.3333, 0.6667, 0.5893)	C-H: 1.110
UDUDUD	*a* = *b* = 2.504; *c* = 15.0	C: (0.3333, 0.6667, 0.5153)	C-C: 1.537
Tricycle	Pbcm (57)	H1: (0.4328, 0.1235, 0.2500)	C1-H1: 1.108
UUUDUD	*a* = 15; *b* = 7.681; *c* = 2.544	C1: (0.4981, 0.0563, 0.2500)	C1-C1: 1.539; C1-C2: 1.541
		H2: (0.6364, 0.1190, 0.2500)	C2-H2: 1.109
		C2: (0.5731, 0.1934, 0.2500)	C2-C2: 1.540; C2-C1: 1.541
Stirrup	Pmna (53)	H: (0.0000, 0.3983, 0.5085)	C-H: 1.105
UUUDDD	*a* = 2.549; *b* = 15.0; *c* = 3.828	C: (0.0000, 0.3639, 0.4620)	C-C: 1.544
Boat-1	pmmn (59)	H: (0.5000, 0.2562, 0.5922)	C-H: 1.105
UUDDUU	*a* = 15.0; *b* = 4.585; *c* = 4.328	C: (0.4622, 0.5939, 0.4317)	C-C: 1.542, 1.548, 1.573
Boat-2	Pbcm (57)	H: (0.3987, 0.4932, 0.5036)	C-H: 1.103
UUUUDD	*a* = 2.529; *b* = 4.309; *c* = 15.0	C: (0.5000, 0.1822, 0.5216)	C-C: 1.537; 1.570
twist-boat	Pcca (54)	H: (0.1215, 0.4079, 0.5609)	C-H: 1.106
UUDUDD	*a* = 4.417; *b* = 15.0; *c* = 4.987	C: (0.0904, 0.4788, 0.6154)	C-C: 1.542; 1.548; 1.562

SG, space group; LC, lattice constant; Position, inequivalent atom positions for H and C atoms; LCH, C-H bond length; LCC, C-C bond length for the six fundamental allotropes of graphane [70].

### Mechanical properties

**Figure 7:**
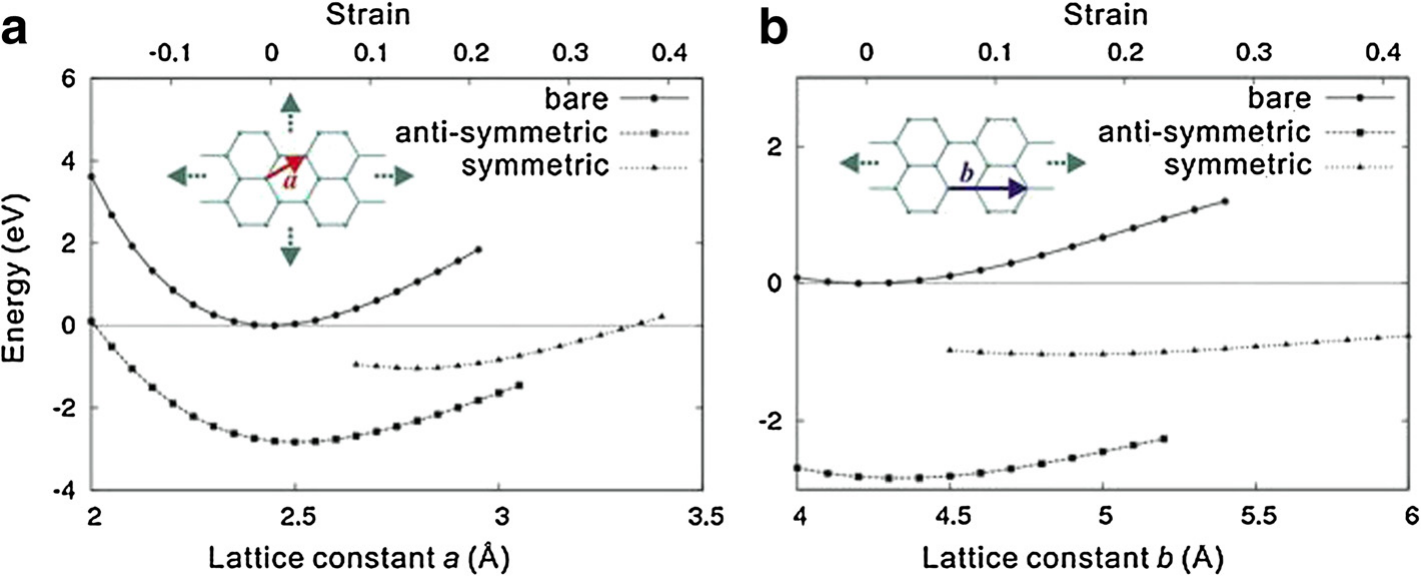
**Energies of pristine graphene.** With additional energy from isolated hydrogen atoms and graphane under **(a)** biaxial and **(b)** uniaxial strain loading [71].

Xue and Xu [71] used a first-principle approach to study strain effects on basal-plane hydrogenation of graphene. Figure 7 shows the predicted energy of both types of graphane structures and also the combined system of pristine graphene and isolated hydrogen atom. The results also show that the in-plane modulus of graphene *C* = *d*^2^*E* / *Adϵ*^2^ = 1,260 GPa is reduced by 52% and 26% in symmetric and antisymmetric phases, respectively, where *E* is the potential energy, *ϵ* is the in-plane biaxial strain, and *A* is the calculated cross-sectional area where the thickness of graphene is taken as 3.4 Å. Accordingly, the biaxial tensile strength has a strong reduction after hydrogenation, from 101.27 GPa to 49.64 and 67.92 GPa due to the hydrogenation-induced rehybridization.

Popova and Sheka [72] used quantum-mechanochemical-reaction-coordinate simulations to investigate the mechanical properties of hydrogen functionalized graphene. Their results showed that the mechanical behavior of graphane was anisotropic so that tensile deformation occurred quite differently along (zg mode) and normally (ach mode) to the C-C bonds chain. The tensile strengths at fracture constituted 62% and 59% of graphene for the ach and zg modes, respectively, while the fracture strains increased by 1.7 and 1.6 times. Young's modules of the two deformation modes of graphane decreased by 1.8 and 2 times. Some mechanical parameters are shown in Table 3.

**Table 3:** **Mechanical parameters of graphene and graphane nanosheets **[72]

**Species**	**Mode**	***ϵ***_**cr**_	***F***_**cr**_**, N (×10**^**-9**^**)**	***σ***_**cr**_**, N/m**^**2**^** (×10**^**9)**^	***E***_***σ*,e**_**, TPa**
Graphene	ach	0.18	54.56	119.85	1.09
	zg	0.14	47.99	106.66	1.15
Graphane	ach	0.3	43.41	74.37	0.61_*σ*_(0.54 e)
	zg	0.23	36.09	63.24	0.57_*σ*_(0.52 e)

Peng et al. [73] investigated the effect of the hydrogenation of graphene to graphane on its mechanical properties using first-principles calculations based on the density functional theory. The results show that graphane exhibits a nonlinear elastic deformation up to an ultimate strain, which is 0.17, 0.25, and 0.23 for armchair, zigzag, and biaxial directions, respectively, and also have a relatively low in-plane stiffness of 242 N/m^2^, which is about 2/3 of that of graphene, and a very small Poisson ratio of 0.078, 44% of that of graphene. There has been a good idea which states that such unique mechanical properties make the graphane a good candidate for materials used in building the tubes or pipelines that transfer materials in high speed under applied high pressure.

### Thermal properties

Ao et al. [74] used the density functional theory to investigate the thermal stability of graphene/graphane nanoribbons (GGNRs). They found that the energy barriers for the diffusion of hydrogen atoms on the zigzag and armchair interfaces of GGNRs were 2.86 and 3.17 eV, respectively, while the diffusion barrier of an isolated H atom on pristine graphene was only approximately 0.3 eV. These results unambiguously demonstrated that the thermal stability of GGNRs could be enhanced significantly by increasing the hydrogen diffusion barriers through graphene/graphane interface engineering. Similarly, Costamagna et al. [75] used large scale atomistic simulations to study the thermal fluctuations of graphane. Rajabpour et al. [76] used nonequilibrium molecular dynamics simulations to investigate the thermal conductivity of hybrid graphene-graphane nanoribbons. Neek-Amal and Peeters [77] used atomistic simulations to determine the roughness and the thermal properties of a suspended graphane sheet. Compared with graphene, graphane had a larger thermal contraction, a wide range corresponding to length scales in the range 30 to 125 Ǻ at room temperature. The estimated heat capacity was 29.32 ± 0.23 J/mol^**.**^K which was 14.8% larger than the one for graphene.

In addition, graphane or graphane-like structures have magnetic properties and thermal performance. Neek-Amal and Peeters [78] investigated the lattice thermal properties of graphane, including thermal contraction, roughness, and heat capacity. Results showed that the roughness, amplitude, and wave lengths of the ripples were very different. The thermal contraction effect of graphane is larger than for graphene. Above 1,500 K, graphane is buckled and starts to lose H atoms at the edges of the membrane. Roughness of graphane is larger than that of graphene and the roughness exponent in graphene decreases versus temperature (from 1.2 to 1.0), while for graphane, it stays around 1.0 implying random uncorrelated roughness. Heat capacity of graphane is found to be 14.8%, which is larger than that of graphene.

### Optical properties

In *Universal optical properties of graphane nanoribbons: A first-principles study* by Yang et al. [78], the results indicated that the optical properties of graphane nanoribbons were independent of their edge shapes and widths. Their unique optical properties make graphane nanoribbons suitable for various applications in nanoscale optical and optoelectronic devices.

### Electronic properties

León and Pacheco [80] studied on the electronic and dynamical properties of a molecular wire consisting of molecules with structures of graphane and a graphane nanoribbon. Bubin and Varga [81] had discussed the response of graphene and graphane fragments to strong femtosecond laser pulses and the results showed that the hydrogenation was controllable by strong femtosecond laser pulses. Before that, Chandrachud et al. [82] had been systematic studied on electronic structure from graphene to graphane. Simultaneously, their results revealed that it was possible to design a pattern of hydrogenation so as to yield a semiconducting sheet with a bandgap much lower than that of graphane. Nanyang Technological University's Hwee Ling Poh et al. [83] investigated the electrochemical behavior of hydrogenated graphene synthesized under various pressures and temperatures for comparison and showed that hydrogenation of graphene (towards graphane) resulting in a decrease in the observed heterogeneous electron transfer rates as measured by cyclic voltammetry and an increase in the charge transfer resistance as measured by impedance spectroscopy as compared to graphene.

### Magnetic properties

Lee and Grossman [84] used the first-principles calculations based on the density functional theory (DFT) to explore the magnetic properties of graphene-graphane superlattices with zigzag interfaces and separately varying widths. The results displayed that the magnetic properties of the superlattices were entirely determined by the graphene region due to the π character of the spin density. It was a potential for future spintronics applications with a variable spin-current density. Berashevich and Chakraborty [85], Schmidt and Loss [86], Şahin et al. [87], and Hernández et al. [88] also did the related research on the magnetism of graphane, such as sustained ferromagnetism, tunable edge magnetism, magnetization of graphane by dehydrogenation, graphane nanoribbons magnetic, and so on.

### Derivatives of graphane

Graphene can be functionalized by varied methods. Haldar et al. [89] used Fe to replace the hydrogen on the plane of graphane. The work showed that the response of the two channels, the armchair and the zigzag channels, were different. Hussain [90] and AlZahrani [91] reported the strain induced lithium functionalized graphane as a high-capacity hydrogen storage material and used the manganese adsorption graphene and graphane as magnetic materials. Graphane's derivatives were not only just about functionalization of the surface atoms, but also by changing the substrate atoms to achieve its function. For example, Lu et al. [41], from the University of Science and Technology of China, studied the chemical modification with –OH or -NH_2_ group on planar polysilane and graphane. Hőltzl et al. [92], Artyukhov and Chernozatonskii [93], Bianco [94], Garcia et al. [95] reported separately in cis-polyacetylene and graphane, carbon monofluoride and graphane, germanium graphane analogue, group-IV graphene, graphane-like nanosheets, and so on.

Therefore, we can fabricate many derivatives of graphane by changing the substrate atoms (like C**,** Si, Ge, P) and the surface atoms (like H, –OH, -NH_2_, He, Li, Fe, Mn, and all the VII A element).

### Applications of graphane

As mentioned in many articles, graphane or graphane-like materials can be applied in many fields. Nechae [96] considered the thermodynamic and experimental backgrounds of the condensed hydrogen storage problem, and an effective method was put forward to produce a high-density hydrogen carrier which was hydrogen intercalation in carbonaceous nanomaterials at relevant temperatures and pressures (at the cost of the hydrogen association energy). The result displayed the intercalated solid molecular hydrogen in graphane-like nanofibers (17 wt.% H_2_). Compared with the US Department of Energy (DOE)'s strategic objectives for the year 2015 which include a minimum 'gravimetric’ capacity (weight of stored H_2_/system weight) of 9.0 wt.% of reversible hydrogen and a 'volumetric’ capacity (density) of 0.081 g(H_2_)/cm^3^(system), graphane-like nanofibers are much more acceptable and efficient hydrogen storage technology.

Gharekhanlou et al. [97] reported that graphane materials can be used as bipolar transistor. Cudazzo et al. [98] provided an exact analytic form of the two-dimensional screened potential. Gharekhanlou et al. [99] introduced a 2D p-n junction based on graphane with hydrogen deficiency to reduce the bandgap effectively. And using basic analysis has shown that within the approximation of Shockley law of junctions, an exponential ideal ***I-V*** characteristic is expectable. This broadens the graphane or graphane-like application in transistor devices. Savini et al. [100] used p-doped graphane to fabricate a prototype high-Tc electron–phonon superconductor, which has Tc as high as 150 K for a 1-nm nanowire, higher than copper oxides. Loktev and Turkowski [101] and Kristoffel and Rägo [102] considered the superconducting properties of multilayer graphane by taking into account the fluctuations of the order parameter. The result showed that in the single-layer case, the BKT critical temperature which corresponds to the vortex SC is equal to the MF temperature 100 K beginning from a rather low value of doping less than 0.01. And they estimated that the critical temperature may reach values 150 K, which is significantly higher than the maximal temperature under ambient pressure in cuprates. Nechaev [103] said that the high-density hydrogen carrier intercalation in graphane-like nanostructures can be used in fuel cell-powered vehicles. Hussian et al. [104,105] used polylithiated (OLi_2_) functionalized graphane as a potential hydrogen storage material, the storage capacity to achieve 12.9 wt.%.

## Conclusions

Exceptional physical properties, chemical tunability, potential electronic, and transistor applications of graphane have definitely gained the interest of materials and electronics researchers. This review article is intended to focus on the fabrication and structural features of graphane (or graphane-like material) and the potential applications of graphane (or graphane-like) and graphane-based nanocomposites. It covers the latest advancement and new perspectives of graphane as a potential material for hydrogen storage and transistor with the special discussions on the merits and limitations of the material. After presenting a brief overview of the synthesis processes of single-layer graphane, graphane-like, graphene-graphane, and graphane nanoribbons, the structure features of graphane, particularly related to the hydrogen storage and transistor, have been discussed.

By reversible hydrogenation, one can make the graphene material from conductor to insulator. Thus, we can control the degree of hydrogenation to modulate the conductive properties. Through this process, graphene-graphane mixed structures offer greater possibilities for the manipulation of the material's semiconducting properties and they can be potentially applied in the field of transistor, electron–phonon superconductor and others applications. The behavior of graphene to graphane or graphane to graphene is the progress of hydrogen energy storage or release. Graphane or graphane-like material can be used as hydrogen storage material for fuel cells. Because of its wide range of conductivity, it can be used for nanosensors with exceptional sensitivity.

Certainly, most notably we can fabricate many derivatives of graphane by changing the substrate atoms (like C**,** Si, Ge, P, S) and the surface atoms (like H, –OH, -NH_2_, He, Li, Fe, Mn, Ag, and all the VII A element) so as to promote its application value and expand the application field.

## Competing interests

The authors declare that they have no competing interests.

## Authors’ contributions

SC and JL designed the structure and modified the manuscript articles; CZ drafted the manuscript. JW, QY, CL, DH, and TZ participated in the sequence alignment. All authors read and approved the final manuscript.
